# Reproducibility and reliability assays of the gene expression-measurements

**DOI:** 10.1186/2241-5793-21-3

**Published:** 2014-05-13

**Authors:** Behrooz Darbani, Charles Neal Stewart

**Affiliations:** Department of Plant and Environmental Sciences, University of Copenhagen, Thorvaldsensvej 40, 1871 Frederiksberg, Denmark; Department of Molecular Biology and Genetics, Research Centre Flakkebjerg, Aarhus University, Forsøgsvej 1, 4200 Slagelse, Denmark; Department of Plant Sciences, University of Tennessee–Knoxville, 252 Ellington Plant Sciences, 37996-4561 Knoxville, TN USA

**Keywords:** Gene expression, Reliability, Reproducibility

## Abstract

**Background:**

Reliability and reproducibility are key metrics for gene expression assays. This report assesses the utility of the correlation coefficient in the analysis of reproducibility and reliability of gene expression data.

**Results:**

The correlation coefficient alone is not sufficient to assess equality among sample replicates but when coupled with slope and scatter plots expression data equality can be better assessed. Narrow-intervals of scatter plots should be shown as a tool to inspect the actual level of noise within the data. Here we propose a method to examine expression data reproducibility, which is based on the ratios of both the means and the standard deviations for the inter-treatment expression ratios of genes. In addition, we introduce a fold-change threshold with an inter-replicate occurrence likelihood lower than 5% to perform analysis even when reproducibility is not acceptable. There is no possibility to find a perfect correlation between transcript and protein levels even when there is not any post-transcriptional regulatory mechanism. We therefore propose an adjustment for protein abundance with that of transcript abundance based on open reading frame length.

**Conclusions:**

Here, we introduce a very efficient reproducibility approach. Our method detects very small changes in large datasets which was not possible through regular correlation analysis. We also introduce a correction on protein quantities which allows us to examine the post-transcriptional regulatory effects with a higher accuracy.

**Electronic supplementary material:**

The online version of this article (doi:10.1186/2241-5793-21-3) contains supplementary material, which is available to authorized users.

## Background

Reliability or accuracy of data-to-reality, and reproducibility or inter-replicate variance, are typically assessed using the correlation coefficient. As a reliability assay, the agreement between transcript abundance and their encoded protein abundance has long been investigated [1–4]. RNA-Seq [5] has made the global profiling of gene expression achievable by covering comprehensive transcriptome data. As the most recent approach, reliability of the output has been examined by correlation analysis using microarray and/or Real Time RT-PCR [5–10]. In addition, the correlation between replicates has been used to judge the reproducibility of data [7, 10–15].

Correlation is the agreement level between two variables. The correlation coefficient varies between perfect agreement (*r* = ±1) and no agreement (*r* = 0). A perfect agreement implies equal change in one of the variables for a given fixed change of another variable. A negative correlation indicates an opposite direction in the agreement, i.e. reduction in one of the variables in response to positive changes in another, regardless of the agreement level. For example, we can find a perfect agreement but in the opposite direction between the dose of insecticides and the number of survived insects.

The inter-replicate variation of gene expression-quantities is of the utmost importance to biologists because lower variance means higher reproducibility. The correlation coefficient has been used to inspect the reproducibility of gene expression data [7, 10–15]. When the inter-replicate variance is low or zero, a higher or perfect correlation is expected but we can find the opposite, i.e. a low inter-replicate variance may be absent when there is a strong correlation. Thus, we cannot rely on the correlation digit as the sole criterion.

Since the correlation coefficient has been the main tool to assess agreement between replicates, technologies (microarray, Real Time RT-PCR, and RNA-Seq), and between protein and transcript abundance, this paper scrutinizes the power of correlation analysis for the biologist and introduces other alternatives. We introduce an alternative reproducibility assay approach. We advise that there should be an application of an inter-replicate based fold-change threshold to identify the most reliable variations among the significant inter­treatment changes regardless of what the inter-replicate reproducibility may be. In addition, we introduce a correction step based upon the lengths of open reading frames (ORFs) for protein quantity before correlation analysis with the respective transcript abundance in order to assess post-transcriptional regulation.

## Results and discussion

### The correlation coefficient is not sufficient as a reproducibility assay for expression data

**Figure 1:**
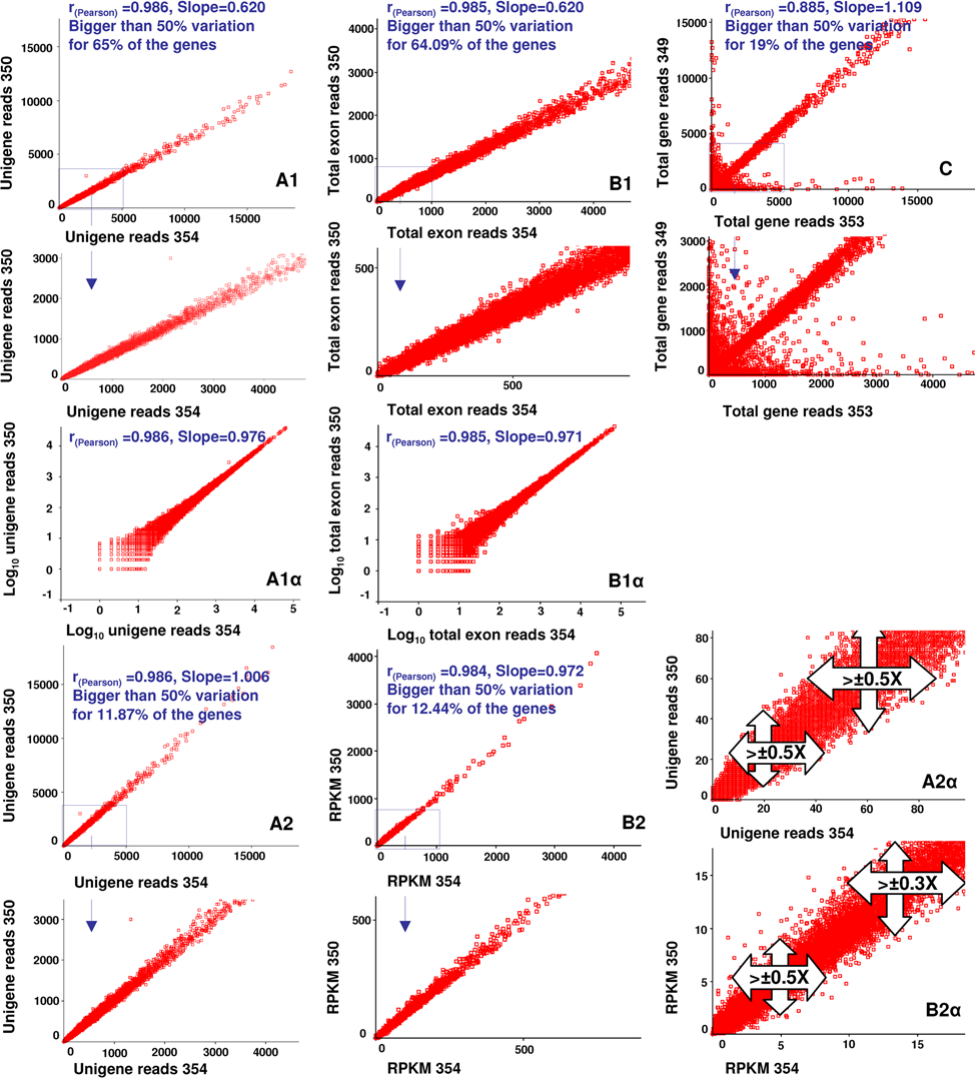
**Correlation-based reproducibility and reliability assays on real gene expression data.** A publicly available RNA-Seq dataset from rice including two root-sample replicates and two shoot-sample replicates is used as an example to illustrate the power of correlation coefficient in the reproducibility assay of read counts as gene expression data. Briefly, the reads were mapped on the rice genome to apply the mapped total gene, unigene, and total exon reads **(A1, A2 and B1, B2)**. Large bias shifts from ~65% in **A1** and **B1** to ~12% in **A2** and **B2** after both reference gene-based correction **(A)** and read count-based correction **(B)** were not able to increase the correlation coefficient. **(C)** As the level of noise increases the correlation becomes weaker. The slopes are nearly the same and approach a slope of 1, but the lower correlation observed results from the higher level of noise in **C** compared to **A2** and **B2**. Considering the level of noise in the data and the slope of inter-replicate regression line, it is possible that, while helpful, they might not be precise for reproducibility assay. After data correction (in **A2** and **B2**), the correlation was not changed but the slope was improved, i.e. the slope-deviation from 1 was decreased. However, 12% of the genes still show 50% or greater than 50% inter-replicate variation in expression, which would be indistinguishable by taking both the correlation and the slope into account. **(A1α, B1α)** Logarithmic transformation of data could change the difference between sample replicates which is represented by the changed slope-deviation on scatter plots. **(A2α, B2α)** Scatter plot narrow-intervals allow us to observe noise in the dataset. There is almost a clear ±0.5x variation shown by scatter plots. 350, 354: rice untreated root sample replicates; 349, 353: rice untreated shoot sample replicates. We evaluated the expression of 24122 to 24701 genes by applying the different types of reads and samples. The log10 transformed data were used to calculate the Pearson correlation. All correlations were significant (*P* = 0.000) by a two-tailed test.

By analyzing a publicly available RNA-Seq data we examined reproducibility of ≈ 25000 genes between two replicates. We found correlations of 0.986 and 0.985 by applying unigene reads and total exon reads, respectively (Figure 1A1 and B1). However, we also found 65% and 64.09% of the genes with a variation higher than 50% between the two replicates (Figure 1A1 and B1). Through further analyses, the insensitivity of the correlation coefficient to the large shifts of inter-replicate variation was confirmed after data correction. Comparisons of Figure 1A1 with A2 and Figure 1B1 with B2 indicate that the large declines in the levels of inter-replicate variances (≈from 65% to 12% of the genes with an inter-replicate variation higher than 50%) were insufficient to influence the correlations. By comparing Figure 1A2 and B2 with 1C where the slopes are nearly the same, it is clear that the correlation coefficient depends on the noise level of the data. Alternatively, relying on the correlation and taking the slope of inter-replicate regression line into account could increase the efficiency of the reproducibility assay. In contrast to the correlation coefficient, considerable improvements of the slopes from 0.620 to 1.006 and 0.972 are good representatives of the correction efficiencies on unigene reads and total exon reads, respectively (Figure 1A1, A2, B1 and B2). However, the threshold level for the noise should be determined by the nature of the experiments. This is crucial when dealing with large sets of data as in microarray or RNA-Seq assays. In this situation, a slope with a small deviation from 1 and a significantly high correlation could coincide with a considerable inter-replicate variation. Although it is not discernible by the slope and the level of noise in the example chosen, 12% of the genes have greater than a 50% inter-replicate variation (Figure 1A2 and B2). Therefore, it is an easy pitfall to ‘neglect’ a few thousand genes with considerable variance among 30,000 genes, which can result in false positive biological conclusions.

Although the actual level of noise on the plots is not visible owing to the large dataset size, the inspection of very narrow intervals of the data range can be helpful (e.g., compare Figure 1A2 and B2 with A2α and B2α, respectively). By following this procedure we found > ±0.5× noise-level in the presented examples, which were not detectable when showing the whole dynamic range of expression. We advise that it would be helpful to include figures and supplements showing narrow-intervals of representations in the reports to provide readers with a useful overall picture.

Scatter plots using the logarithmic scale have been reported previously to represent expression data reproducibility [2, 4, 5, 11, 12, 15–17]. However, logarithmic transformation of data evokes changes in the variation between replicates, which affects slope and diminishes its usefulness. The similarity level between replicates and the percent of data which are 1 and lower than 1 have an impact on the direction, i.e. whether slope-deviation will be increased or decreased, and the rate of change of slope. We found considerable decreases in the deviation of the slope of inter-replicate regression line using uncorrected data where the inter-replicate similarity levels were low (compare Figure 1A1 and B1 with A1α and B1α, respectively). This artificially low slope-deviation could be incorrectly interpreted as high reproducibility. In conclusion, logarithmic scale slope is of little utility to reproducibility assays.

To examine the reliability of gene expression measurements, the agreement levels of expression data between technologies, i.e. RNA-Seq, microarray, and Real Time RT-PCR, have also been investigated [5–10]. These assays might have general agreement with each other but equality is not observed. Therefore, we should require a strong positive correlation but not equality among platforms, which could be represented by the slope of inter-replicate regression line. Aside from the evidence-free acceptance of one technology as the most reliable one, reliability assays suffer from the inefficiency of correlation coefficient when analyzing large sets of data. The acceptable threshold of correlation should be evaluated. Like for the reproducibility assay, we advise that the real level of noise should be examined on narrow-intervals of data ranges in scatter plots.

### The mean and standard deviation of the inter-treatment gene expression ratios shed light on reproducibility

As an alternative to the correlation coefficient, we advise reporting the mean and standard deviation of the inter-treatment gene expression ratios to examine reproducibility. By calculating the ratios of each of the descriptive statistics for single replicates when compared to different replicates of another treatment, we should check the deviation of ratios from 1. The average of deviations and their standard deviation could be utilized as reproducibility coefficients. The higher deviation and higher standard deviation for the deviations indicate lower reproducibility.

As an example, we performed the analysis on the total exon mapped reads for a rice experiment and its corrected “reads per kilobase of exon model per million mapped reads” (RPKM) data by applying different inter­treatment comparisons of untreated root and salinity treated shoot, untreated shoot and salinity treated shoot, untreated shoot and untreated root, untreated root and salinity treated root, salinity treated root and salinity treated shoot, and salinity treated root and untreated shoot (see Additional file 1). Analysis of two replicates of each treatment resulted in 2 × 48 ratios for each of the datasets, i.e. corrected and uncorrected reads. The average of the ratios’ deviations were 0.338 and 0.123 and the standard deviation of ratios’ deviations were 0.318 and 0.133 on total exon reads and on RPKM data, respectively. As expected, these coefficients represent higher reproducibility when using the corrected RPKM data. Compared to the correlation-based reproducibility assay, where there was no difference between the correlations of uncorrected and corrected data, it is also more effective to examine reproducibility by applying the new approach. As shown in Figure 1, the correlation coefficient was not effective to represent the significant reduction in the inter-replicate variance (≈from 65% to 12% of the genes with an inter-replicate variation higher than 50%) after data correction. In contrast, the reduction from 33.8% to 12.3% in the reproducibility level proves the effectiveness of the new method compared to the correlation coefficient. Therefore, the new approach provides a higher level of efficiency to represent different variability levels compared with the correlation-based approach, at least using the applied experimental data. In addition, the decreased standard deviation of ratios’ deviations from 0.318 to 0.133 indicates an increased reproducibility among the treatments after data correction. A similar approach is also applicable between replicates just by applying the deviations of averages and standard deviations of gene expression ratios. The average and standard deviation of inter-replicate expression ratios are expected to be 1 and 0 when there is perfect reproducibility. However, we need a higher number of replicates to obtain reliable measures. This should not be a concern when the main aim is to evaluate the reproducibility of a method or technology. As shown by the example analyzed here, four samples each with two replicates works well. However, it is worth to evaluate the impact of the number of replicates on the proposed method. Our analysis indicates that the method works at an acceptable level on a dataset with three replicates. By analyzing an online-available yeast RNA-Seq dataset [18] we found a Pearson correlation of 0.994 among technical replicates of samples 1 and 2 while 4.3% and 4.2% of the 6387 genes had 50% or higher inter-replicate variations, respectively. In contrast, our proposed approach found 14.2% and 13.7% deviations for samples 1 and 2, respectively. This indicates that the proposed method can inform small changes, e.g., 4.3% to 4.2% of the genes with 50% or higher inter-replicate variations. In agreement with this, we also found a 15.3% deviation when comparing biological replicates, i.e. sample 1 with sample 2, with 4.4% of the genes showing 50% or higher inter-replicate variations (see Additional file 1). This indicates that our method has high sensitivity and can even detect very small changes in large datasets. Therefore, we advise our method to be utilized as an efficient alternative to the correlation coefficient. To examine the efficiency of our method on various gene expression datasets, we analyzed microarray data from *Arabidopsis*, barley, yeast, and *E. coli* (Table 1; Additional file 2). Similar to the RNA-Seq data, correction of the microarray data resulted in strong reduction in the number of genes showing over 50% inter-replicate variations without considerable improvement in the correlation coefficients between replicates (Table 1). The average of slopes of inter-replicate regression lines showed improvements after data correction (Table 1) and as discussed before, it can complement the correlation coefficient. As seen in RNA-Seq data, very high correlations (>0.98) and small slope deviations (<0.02) are possible while up to 10% of the genes show over 50% inter-replicate variations in microarray data (Table 1). In addition, not only do the slopes not show any considerable improvement after correction of the *E. coli* microarray data, but they also decreased while the number of genes with higher than 50% inter-replicate variation was decreased from 8-10% to 2.9% (Table 1). In contrast, our new measurements followed the increased reproducibility and showed considerable reduction in deviations after data correction (Table 1; Additional file 2). However, the deviations were still large enough to explain the existent variance after data correction in all the experiments.

**Table 1:** **Reproducibility of the microarray data**

	Uncorrected data				After quantile correction			
Experiment^***a***^	Average number of genes with >50% inter-replicate variation	***r***_(P)_^***b***^	Slope^***c***^	A.D^d^ %	Average number of genes with >50% inter-replicate variation	***r***_(P)_^***b***^	Slope^***c***^	A.D^d^ %
**E-GEOD-53990:** ***Arabidopsis*** **data with 6 treatments and 3 replicates**	8488/22810 (37%)	0.975	0.804	91%	2000/22810 (8.8%)	0.980	0.982	26%
**E-GEOD-27822: Barley data with 2 treatments and 3 replicates**	6433/22840 (28%)	0.992	1.22	34%	519/22840 (2.3%)	0.994	0.992	8.2%
**E-GEOD-39950: Yeast data with 2 treatments and 3 replicates**	1645/10928 (15%)	0.997	1.257	26%	396/10928 (3.6%)	0.997	0.980	12%
**E-GEOD-49918:** ***E. coli*** **data with 4 treatments and 3 replicates**	1059/10208 (10%)	0.996	1.032	28%	299/10208 (2.9%)	0.997	0.981	10%
**E-GEOD-24524:** ***E. coli*** **data with 4 treatments and 2 replicates**	837/10208 (8.2%)	0.995	0.999	22%	300/1028 (2.9%)	0.996	0.990	15%

Different publicly available microarray data were analyzed using the Affymetrix Expression Console build 1.3.1.187 following the 3 Expression Arrays-RMA protocol. Both the corrected and uncorrected data were extracted. The Pearson correlations between the replicates were calculated on the log_2_ transformed data. Following our method, the average of the ratios’ deviations was also calculated in order to evaluate its usefulness compared to the correlation coefficient.

^*a*^The experiments are available at http://www.ebi.ac.uk/arrayexpress/experiments/browse.html, ^*b*^The average of Pearson correlations between the replicates in each experiment, ^*c*^The average of slopes of inter-replicate regression lines in each experiment, ^*d*^The average of the ratios’ deviations.

It should be emphasized that the levels of reproducibility should not be compared among different experimental data. This is due to the data-dependency of the measured statistics. We found higher deviation values (15.3%, 14.2%, and 13.7%) for the yeast data when there were fewer genes with greater than 50% inter-replicate variance (4.4%, 4.3%, and 4.2%) compared to the rice data.

As indicated, we have included X/Y and Y/X as separate measurements of the genes between the treatments in order to fix the asymmetric aspect of the ratios around 1. We have not applied the well-known logarithmic transformation, i.e. log (X/Y) = log (X) – log (Y). The latter, can easily reduce the difference level between comparisons as we have already explained. Therefore, we will not see the actual difference at the reproducibility levels by applying the logarithmic transformation.

### What can be done when reproducibility is low or not known?

The correlation coefficient or the alternative method introduced here shed light on general data reproducibility based on predefined thresholds. The main application of these approaches is in comparing different analytical methods such as correction measures with each other in order to use the most appropriate method. However, they do not provide detailed information as to which part of data has an acceptable reproducibility or what stringency level we should apply in our analysis to meet the low reproducibility levels in order to avoid making the data useless. Under the assumption of a similar natural variation of expression for all genes, i.e. since a gene with extreme inter-replicate variation for expression is assumed to be random, we advise applying a fold-change threshold to solve the problem. To be acceptable, the level of the statistically significant expression changes between the treatments should be not smaller than the predetermined threshold. This threshold should be determined by keeping the frequency of genes showing same or higher levels of inter-replicate changes lower than an acceptable likelihood of being false positive, i.e. 5% or 1%. Therefore, a significant inter-treatment fold­change of a gene should have an inter-replicate likelihood lower than either 1% or 5%. Applying this criterion keeps the usability gate open even when the reproducibility is low just by considering the reliable fold-changes. When analyzing the rice untreated root [GenBank ID: DRR000350 and DRR000354] and untreated shoot [GenBank ID: DRR000349 and DRR000353] samples, we found the fold-­change thresholds of >3.7 and >2.0 at 1% and 5% levels, respectively. By comparing untreated shoot with untreated root, we found 5212 (48% of the significant differentially expressed genes) and 783 (7.3% of the significant differentially expressed genes) genes showing same or lower levels of fold-changes according to the 1% and 5% cut-offs, respectively. We can also apply a gene­group specific fold-change threshold when there is a bias towards specific genes, e.g., by gene-length or expression-quantity based clustering of the genes due to the higher variation of short­length or low-abundant genes compared to the long or high abundant genes, respectively, in RNA-Seq.

### ORF length-based correction on protein quantities is necessary before correlating them with their transcript abundance

No strong correlation has been found between protein and the RNA abundance [1–4]. One reason could be the multiple levels of regulatory mechanisms, from transcription to post-translation, that govern the expression of genes. By excluding all the technical and biological noise as well as experimental biases, different determinants including ribosomal occupancy (the fraction of a given gene’s transcripts engaged in translation at least by one ribosome) and ribosomal density (number of ribosomes per length-unit of transcripts) as translation activating factors, sequence features of translation initiation, elongation, and termination as ORF-specific translation-efficiency related factors, and amino acid availability as well as mRNA/protein half-life have been proposed to influence the mRNA-protein correlation [19–22]. Among these, ORF-specific translation-efficiency related factors explain 15-30% of the variation of mRNA-protein correlations [22–24]. The mRNA half-life does not show a considerable impact in contrast to the protein half-life, which explains ≈ 17% of the mRNA-protein correlation variations [24].

For many reasons, however, biologists most typically rely on transcript abundance as a gauge for gene expression. But, how reasonable is it to expect a high correlation between the transcript and the protein levels? To answer, let us assume the two expressed genes, A and B, are governed by post-transcriptional-free regulation mechanisms. This means that they have the same stability, half-life, and translation efficiency for the transcripts and same stability and half-life for the proteins of genes A and B. It is still possible to have different rates of induction or suppression between the protein levels of genes when there is a similar induction or repression rate at the transcript levels of the genes. Similar rates of change at the transcript levels of the genes A and B could result in different rates of change at the protein levels when the lengths of the coding sequences differ between the genes. It might be expected to find twice as much induction for the protein A compared to the protein B if the coding sequence of gene B is twice as long as that of the gene A. The longer protein B will take more time to be translated than the protein A. This can simply decrease the correlation between the protein and the RNA levels by uneven variation of the variables (the transcript level and the protein level). As an example, the induced protein levels for A and B were four and two times, respectively, if the transcript levels of the genes were induced two times. In this situation, there are uneven changes of two and four times at the protein levels (one of the variables) compared to a fixed two times change in transcript levels (another variable). Therefore, it is not reasonable to look at the agreement level between the RNA and their encoded protein quantities. Instead, we should consider the effects of treatments/tissues on the gene expression quantities regardless of gene expression assay. In conclusion, different levels of post-transcriptional regulations should not be interpreted as the sole reason for the medium-weak correlation between the protein and transcript levels due to the above-mentioned technical problem which could easily have a large impact on the issue, either positively or negatively.

To inspect the pure effects of post-transcriptional regulation we advise that protein quantities should be corrected for the length of coding sequences before correlating the protein and the transcript quantities with each other. We performed a length-based correction on the previously reported protein quantities before examining the correlation between protein and transcript quantities. For the 10 proteins of *Plasmodium falciparum* and their corresponding transcript abundance measured during six growth stages published in [2], the correlation showed a shift from −0.029 to 0.135 after the ORF length-based correction was applied for protein quantities (Additional file 1). We also examined two different expression datasets (Additional file 1): cell cycle with 173 genes and cell rescue with 115 genes in yeast where the expression quantities exist both at the protein and at transcript levels, reported by Greenbaum *et al.*[1]. After the length-based correction on protein quantities the Pearson correlations were changed from 0.71 to 0.41 and from 0.45 to 0.29 in cell cycle and cell rescue datasets, respectively.

To further analyze the impact of ORF length-based bias in quantitative analysis of transcript and protein expressions, we looked at the correlation of mRNA changes with protein changes between human and chimpanzee reported by Fu *et al.*[25]. We do not expect a negative correlation between changes of transcripts and of proteins. This is due to the fact that cells try to consume as little energy as possible. In addition, an overall trend of decreased mRNAs and increased proteins is not reasonable. However, we found a correlation of −0.37 between mRNAs (chimpanzee/human ratios) and proteins (chimpanzee/human ratios) of 143 genes. The averages of five and three biological replicates were applied to calculate the mRNAs changes and the proteins changes, respectively (Additional file 1). In contrast, a more reasonable correlation of −0.11 was obtained by using the ORF length-based corrected protein changes (Additional file 1).

Increased ribosomal occupancy means increased usage of minimum physical space on transcripts for ribosomal functionality. This can alleviate the effect of different ORF lengths on the deviations of fold-changes of protein quantities from the fold-changes of transcript quantities. In agreement with this, a higher protein transcript correlation was reported for the transcripts with high ribosomal occupancy [1]. This indicates that the ORF length differences can easily affect the agreement between the transcripts and their coded protein quantities.

It is conceivable that the longer transcripts may be occupied by a higher number of ribosomes, which might alleviate the aforementioned effect of the ORF length on the correlation coefficient between protein and transcript abundance. However, the very weak correlation (*r*_P_ = 0.22, *r*_S_ = 0.34) between the ORF length and number of ribosomes on the transcripts and negative­weak correlation (*r*_P_ = −0.39, *r*_S_ = −0.56) between the ORF length and ribosomal occupancy, which we found (see Additional file 1) in the data published by Greenbaum *et al.*[1], introduce the number of ribosomes or the ribosomal occupancy of transcripts not as a random length-based event, but rather, as a post-transcriptional regulation mechanism. Not only there is no strong correlation between the transcript length and ribosome numbers but also longer transcripts show lower ribosomal density compared to shorter transcripts reported in [19]. This could be explained by the fact that the first 40 codons of transcripts have over three-fold higher ribosomal density compared with any given 40 codons long fragment from the +40th codon downstream region of transcripts [26]. In addition, trans-regulatory divergence and not ribosomal occupancy has recently been introduced as the force behind the differences in the yeast translational efficiency [27]. Therefore, there should not be any concern about the alleviated effect of ORF length on the correlation coefficient. Correction for ORF length on protein quantity seems to be useful to extract much pure post-transcriptional regulation effects, at least based on the available data.

Although we introduce ORF length as a factor affecting the mRNA-protein correlation, it should be highlighted that our ORF length-based correction of protein quantities is too simplistic and cannot guarantee the purity of the variation of mRNA-protein correlation. Among the ORF-specific translation-efficiency factors, peptide elongation has been found as the major determinant to explain the variation of mRNA-protein correlation [22–24]. One conceivable reason could be the biological importance of the elongation process itself compared to the initiation and termination processes. However, the other key player could be the transcript/ORF length, which can easily influence the elongation process. Therefore, our introduced ORF length-based correction of protein quantities can be affected by the elongation-related regulatory variance and needs further development in order to extract the pure length-dependent variance from the variation of mRNA-protein correlation.

## Methods

### RNA-Seq data analyses

To evaluate the efficiency of correlation coefficient on real data, a publicly available rice RNA-Seq dataset was used in the analysis. The dataset included two replicates of root and shoot samples from plants before and after a one hour salinity stress treatment [GenBank ID: DRX000191, DRX000192, DRX000193, and DRX000194]. The software CLC Genomics Workbench (http://www.clcbio.com; Aarhus, Denmark) was used to map the reads on the rice Nipponbare reference genome to isolate different type of reads, i.e. total and uniexon and gene reads. The average of inter-replicate variations of selected reference genes were applied for the reference gene based data correction (data not shown). The differentially expressed genes were found as described by Robinson *et al.*[28] with a *p*-value cut-off of 0.01 and using the Benjamini & Hochberg method [29] for multiple testing correction.

### Microarray data analysis

We also analysed four different microarray datasets which are publicly available in EMBL-EBI, ArrayExpress (http://www.ebi.ac.uk/arrayexpress/). The datasets studied included *Arabidopsis* data [E-GEOD-53990] with six treatments and three replicates, barley data [E-GEOD-27822] with two treatments and three replicates, yeast data [E-GEOD-39950] with two treatments and three replicates, and two *Escherichia coli* datasets [E-GEOD-49918 and E-GEOD-24524]. One *E. coli* dataset had four treatments and three replicates and another had four treatments and two replicates. We used the Affymetrix Expression Console build 1.3.1.187 to analyze the data; the 3 Expression Arrays-RMA was applied to extract the quantities both without any correction and together with quantile-based correction.

### An alternative reproducibility test by measuring the mean and standard deviation statistics of inter­treatment ratios of gene expression quantities

After measuring the descriptive statistics of mean and standard deviation for inter­treatment expression ratios, we calculated the ratios of each statistic for a single replicate when compared to different replicates of another treatment. Perfect agreement between replicates would result in a ratio of 1. Therefore, any deviation could be an indicator of decreased reproducibility. The analytical procedure was as follows:

1. Measurement of the genes’ expression ratios between the treatments
  T_1iR(T1)_/T_2iR(T2)_, T_1iR(T1)_/T_3iR(T3)_, T_2iR(T2)_/T_3iR(T3),_ …, T_n-1iR(Tn-1)_/T_niR(Tn)_, and T_2iR(T2)_/T_1iR(T1)_, T_3iR(T3)_/T_1iR(T1)_, T_3iR(T3)_/T_2iR(T2)_…, T_niR(Tn)_/T_n-1iR(Tn-1)_
  where T represents the treatments (1,…, n) and i represents the genes. R_(T1)_, …, and R_(Tn)_ represent the replicates of treatments.
2. Computing the mean (M) and standard deviation (S) for each population of ratios
  ^M^T_1iR(T1)_/T_2iR(T2)_, …, ^M^T_n-1iR(Tn-1)_/T_niR(Tn)_, and T_2iR(T2)_/T_1iR(T1)_, …, ^M^T_niR(Tn)_/T_n-1iR(Tn-1)_
  ^S^T_1iR(T1)_/T_2iR(T2)_, …, ^S^T_n-1iR(Tn-1)_/T_niR(Tn)_, and ^S^T_2iR(T2)_/T_1iR(T1)_, …, ^S^T_niR(Tn)_/T_n-1iR(Tn-1)_
3. Computing the ratios of the statistics between paired populations among which one of the replicates of one sample is compared to all the replicates in another sample.
  For example, in case of ^M^T_1iR(T1)_/T_2iR(T2)_ we do as follows:
  [^M^T_1i1_/T_2i1_]/[^M^T_1i1_/T_2i2_], [^M^T_1i1_/T_2i1_]/[^M^T_1i1_/T_2i3_], …, [^M^T_1i2_/T_2i1_]/[^M^T_1i2_/T_2i2_], [^M^T_1i2_/T_2i1_]/[^M^T_1i2_/T_2i3_],
  The same calculation should be performed for the ratios of standard deviations. Perfect agreement between replicates would result in a ratio of 1 and deviations can be an indication of decreased reproducibility.
4. Measuring the variation of the ratios
  Since we deal with ratios, the variations should be applied in order to calculate the deviations from 1. For that, we must use the ratios of X/Y when X > Y. If X < Y, then we must use 1/(X/Y).
5. Measuring the average and standard deviation of the deviations of ratios
  These parameters could be utilized as reproducibility coefficients. There will be no deviation when there is a perfect reproducibility. In addition, the standard deviation of the deviations is an indication of how constant the reproducibility is when considering all treatments. To facilitate all the steps of the analyses, an Excel file (Additional file 3) is prepared for users. It includes different sheets based on the number of treatments and replicates and it just need the data to be pasted in in order to calculate the reproducibility measurements. There is a manual sheet included as well. We analyzed the mentioned rice data including four treatments each with two replicates. Therefore, there were 48 populations of expression ratios for uncorrected data and the same for corrected data (see Additional file 1). To check the robustness of the method, we also applied the online-available yeast RNA-Seq dataset explained by Lipson *et al.*[18]. This includes expression data of 6387 genes reported as transcript per million (t.p.m) and were found expressed in all the three technical replicates of the samples (see Additional file 1). Furthermore, we analyzed the mentioned five different microarray datasets from *Arabidopsis*, barley, yeast, and *E. coli* (see Additional file 2).

### Protein quantity correction in order to assess the correlation between the protein and transcript abundance

Translation time can vary among transcripts with different lengths. This could be a potential source of variation of mRNA-protein correlation. Here, we introduce a correction approach to expel this source of variation out of data. Table 2 represents an example of protein quantity correction. The ORF lengths could be corrected by dividing the ORF-of interest-length by the shortest ORF length in the data. The corrected lengths are the correction factors that act as multipliers on protein quantities prior to correlating them with respective transcript abundance (Table 2).

**Table 2:** **Pure post-transcriptional regulation effects can be examined after correction of the protein quantities**

Gene	TF^***a***^	ORF^***b***^(bp)	PF^***c***^	CF^***d***^	CPF^***f***^
1	2	1000	10	1	10
2	2	2000	9	2	18
3	2	3000	8	3	24
4	2	4000	7	4	28
5	2	5000	6	5	30
6	2	6000	5	6	30
7	2	7000	4	7	28
8	2	8000	3	8	24

The table represents an artificial example of similar transcript fold-change for all the genes with different lengths and different protein fold-changes.

^*a*^Observed transcript fold-change, ^*b*^Coding sequence length, ^*c*^Observed protein fold-change, ^*d*^Calculated correction factor (length/1000), ^*f*^Corrected PF (PF.CF).

## Conclusions

Here, we conclude to enhance the power of reproducibility assay of gene expression data by considering the slopes of inter-replicate linear regression lines and narrow-intervals of scatter plots as well as the correlation coefficient. We also introduce an efficient alternative approach which can detect small changes among large datasets. This works based on the means and standard deviations of the expression ratios of genes. As a reliability assay, it is advised not to rely on the correlation between the transcript and protein levels. This is due to the differences among the ORF-lengths of genes which can result in a weak correlation without post-transcriptional regulatory mechanisms. Finally, we advise an ORF-length based correction of protein quantities to examine a much pure effect of post-transcriptional regulatory mechanisms.

## Electronic supplementary material

Additional file 1: **It is an Excel file which contains all RNA-Seq data analyses plus RNA-protein datasets.** (XLSX 4 MB)

Additional file 2: **It is an Excel file which contains all the microarray data analyses.** (XLS 2 MB)

Additional file 3: **This Excel file can be used as an easy tool in order to get the reproducibility measurements.** (XLS 268 KB)
